# Editorial: Recent Advances in Bovine Tuberculosis

**DOI:** 10.3389/fvets.2022.907353

**Published:** 2022-04-26

**Authors:** Federico Carlos Blanco, Christophe J. Queval, Flabio R. Araujo, Jacobus Henri De Waard

**Affiliations:** ^1^CICVyA, Institute of Biotechnology, National Institute of Agricultural Technology (INTA), Buenos Aires, Argentina; ^2^HTS, Francis Crick Institute, London, United Kingdom; ^3^Brazilian Agricultural Research Corporation (EMBRAPA), Brasília, Brazil; ^4^One Health Research Group, Facultad de Ciencias Veterinarias, Universidad de Las Américas, Quito, Ecuador

**Keywords:** *Mycobacterium bovis*, bovine tuberculosis, one health, diagnostic, immunology, vaccination, epidemiology

According to the World Organization for Animal Health (OIE), bovine tuberculosis (bTB) is a chronic infectious disease in animals caused by members of the *Mycobacterium tuberculosis* complex, and in most cases by *Mycobacterium bovis* (Mb) (1). It is a zoonotic disease, where cattle are the main source of infections for humans. Mb also affects other animals such as goats, sheep, cats, and dogs, as well as wild and zoo animals (2). Moreover, other species are recognized as Mb maintenance hosts in several countries: wild boar in Iberia, the European badger in Great Britain and Ireland, the African buffalo and Marsh antelope in Africa, brushtail possum in New Zealand, and white-tailed deer in the USA (3). The eradication of the disease is a major problem and policies of surveillance and control have been applied in several countries using screening and stamp-out strategies. As part of the One Health concept, bTB is a priority disease and all efforts to eradicate bTB should be compulsory (4). However, the policies enacted to eliminate this disease can have serious financial impacts on farmers. Therefore, one of the most challenging tasks in eliminating bTB is to provide accessible solutions at an affordable cost that can easily be implemented in developing countries where, in general, the prevalence of bTB in animals and humans is high.

This special issue of Frontiers in Veterinary Science is centered on the latest discoveries made in the field. It comprises 16 articles reporting on epidemiology, diagnosis, vaccination, pathology, and host-pathogen interactions and Immunology. The majority of the articles are focused on cattle. However, there are three major studies involving other hosts of Mb; goats, buffaloes, and deer. Concerning goats, the implementation and advances of the diagnosis with serology in oral fluid are discussed (Ortega et al.). The use of overlapping peptides representing the ESAT-6, CFP-10, and Rv3615c antigens in single intradermal skin test (SIT) has been used for diagnosis in buffaloes by Kumar et al. In Ireland, sika deer have been identified as a reservoir for bTB and should be considered an integral part of the bTB-control program (Kelly et al.). The zoonotic transmission of Mb from cattle to humans has been recognized for a long time, but transmission from humans to cattle is less often recognized. In this special issue, there is a report of two human/cattle transmission cases in North Dakota, USA (Lombard et al.).

Relating to the diagnosis, the oldest and most used method for bTB diagnosis is the intradermal skin test (Good et al.). Its main disadvantages are the variable sensitivity and specificity, as shown in several publications (5). Moreover, tuberculosis vaccination strategies interfere with this test as animals get sensitized, causing false positives (6). The interferon-gamma release assay (IGRA), based on the quantification of this cytokine in the supernatant of whole blood cultures, stimulated *in vitro* with Mb antigens, has been approved in several countries as a complementary diagnostic method (7). Different antigens can be used for the *in vitro* stimulation, wherein purified protein derivate (PPD) and peptide cocktails to acquire a higher specificity are the most common ones. In this special issue, investigators from Switzerland evaluated the specificity of three commercial IGRAs in different cattle breeds from bTB-free herds (Ghielmetti et al.).

Molecular techniques for detecting the genetic material of Mb have been developed and are used for diagnosis but also for the epidemiology of bTB. These techniques can be applied in order to detect Mb in blood, tissue, or milk and its derivatives. In one of the articles presented in this collection, researchers from Spain applied Real-time PCR (qPCR) for the direct detection of Mb in lymph nodes from carcasses obtained at the slaughterhouse. The agreement between microbiological culture and this qPCR was almost perfect (Sánchez-Carbajal et al.). Another article in this collection used whole-genome sequencing (WGS) and characterized the diversity of Mb genotypes circulating in Baja California, Mexico, with Mb isolates recovered from humans, cheese, and cattle, providing strong evidence of human-to-cattle-to-cheese transmission (Ortiz et al.). WGS, together with Single Nucleotide Polymorphism (SNP)-analysis, was also applied in the Brazilian Amazon region to analyze Mb inter-species transmission between cattle and buffaloes. Additionally, a new Mb lineage (Lb1) for South America was detected (Carneiro et al.).

Acknowledging the distribution, population structures, and transmission networks of the different Mb isolates in every region of the world is crucial for identifying the source of bTB infection, its transmission dynamics and host preference, the influence of wildlife reservoirs on transmission to cattle, and thus the implementation of effective strategies to contain it. For the genotyping of Mb, the spoligotyping technique is currently considered the gold standard. Nonetheless, this a lengthy and technically demanding method. In an article from Brazil, an alternative method for the genotyping of Mb is presented: Multispacer Sequence Typing, based on sequencing. This is more accessible for most laboratories (Bravo Sales et al.). Spoligotyping and 24 loci MIRU-VNTR typing were used in an article from the state of Pernambuco, Brazil, showing high variability of Mb strains in this region (30 strains and 15 different genetic profiles), indicating several introductions of Mb strains and consequently a failing TB control program (Melo et al.).

In this special issue, the virulence of Mb has been addressed with molecular biology techniques for a better understanding of the functional impacts of the nucleic acid differences between Mb and *M. tuberculosis* (Mt). Whole-genome transposon libraries in laboratory strains of both species were generated and the essentiality status of genes during growth under identical *in vitro* conditions was assessed (Gibson et al.). For growth, 527 genes were found to be essential in Mb, whereas 477 genes were essential in Mt and 370 essential genes were common in both species.

Concerning the vaccination of cattle, an interesting article is a meta-analysis by experts from India, the USA, the United Kingdom, the Netherlands, and Ethiopia, which evaluates the effect of BCG vaccination (Srinivasan et al.). Their analyses suggest that BCG vaccination may help to accelerate the control of bTB in endemic settings.

Last but not least, the immunology of the Mb infection has been addressed in five articles. Bovine TB pathology is characterized by pulmonary and lymph node lesions, subsequently leading to granuloma formation. Both the chronic evolution and immunopathology of bTB resemble human TB in several aspects. In this particular collection, we have two major studies that describe the evolution of bTB lesions and immune response in a tme-course manner. A group from the USA examined bacterial burden and cytokine expression in individual pulmonary granulomas from steers at 30, 90, 180, and 270 days after experimental aerosol infection with Mb (Palmer et al.), concluding that bacterial burdens did not correlate with the expression of IFN-γ, TNF-α, TGF-β, granuloma stage, or lung lesion score, although there was a modest positive correlation with IL-10 expression. In another study, the transcriptome of bovine whole peripheral blood samples collected at pre-infection and up to 12 weeks post-infection time points were analyzed for promising gene expression biomarkers for bTB. These authors identified a 19-gene transcriptional biosignature of infection consisting of genes increased in expression across the time course of 12 weeks post-infection with Mb (McLoughlin et al.).

Cell-mediated immunity plays an important role in the control of the Mb infection, and the characterization of such responses is important for understanding the immune status of the host and identifying mechanisms of protective immunity or immunopathology. Researchers from the USA analyzed the T-cell response following experimental *M. bovis* infection in cattle *via in vitro* antigenic expansion and re-stimulation to characterize antigen-specific CD4, CD8, and γδ T cells and their functional phenotype, shedding light on the variable functional ability of these cells. These data can help us better understand the cellular response to Mb infection and develop improved vaccines and diagnostic tools (Boggiatto et al.).

Despite the high degree of identity that Mt and Mb share both at the genetic level as well as during the infection process, the two pathogens display distinct tropism and virulence depending on the host. While Mb is highly virulent and pathogenic for cattle and a range of other mammals, Mt is mostly restricted to humans. From France and Ireland, there is a study in this special issue that investigated the bovine innate response following Mb or Mt infection in a lung slice model of two local cattle breeds. A striking difference in the early lung response to Mb infection was found between these two breeds. The transcriptomic signature induced by infection was very low for the Charolaise breed, whichever mycobacterial strain was used, pointing to the possible control of Mb infection at the genetic or epigenetic level. In the other breed, the type 1 interferon pathway was only induced by Mb infection but not Mt infection (Remot et al.).

In conclusion, in this special issue of Frontiers in Veterinary Science, you can find articles summarizing the recent advances in tackling bTB. This special issue presented by leading researchers in the field is a great opportunity to update your knowledge concerning the control, diagnosis, immunology, and epidemiology of this infectious disease.

## Author Contributions

All authors listed have made a substantial, direct, and intellectual contribution to the work and approved it for publication.

## Conflict of Interest

FA was employed by Brazilian Agricultural Research Corporation. The remaining authors declare that the research was conducted in the absence of any commercial or financial relationships that could be construed as a potential conflict of interest.

## Publisher's Note

All claims expressed in this article are solely those of the authors and do not necessarily represent those of their affiliated organizations, or those of the publisher, the editors and the reviewers. Any product that may be evaluated in this article, or claim that may be made by its manufacturer, is not guaranteed or endorsed by the publisher.
